# Pangenomic Study of Fusobacterium nucleatum Reveals the Distribution of Pathogenic Genes and Functional Clusters at the Subspecies and Strain Levels

**DOI:** 10.1128/spectrum.05184-22

**Published:** 2023-04-12

**Authors:** Xiaomei Ma, Tianyong Sun, Jiannan Zhou, Mengfan Zhi, Song Shen, Yushang Wang, Xiufeng Gu, Zixuan Li, Haiting Gao, Pingping Wang, Qiang Feng

**Affiliations:** a Department of Human Microbiome & Implantology & Orthodontics, School and Hospital of Stomatology, Cheeloo College of Medicine, Shandong University & Shandong Key Laboratory of Oral Tissue Regeneration & Shandong Engineering Laboratory for Dental Materials and Oral Tissue Regeneration, Jinan, China; b The State Key Laboratory Breeding Base of Basic Sciences of Stomatology, Key Laboratory of Oral Biomedicine, Ministry of Education (Hubei-MOST KLOS & KLOBM), School and Hospital of Stomatology, Wuhan University, Wuhan, China; c State Key Laboratory of Microbial Technology, Shandong University, Qingdao, China; Tianjin University

**Keywords:** *Fusobacterium nucleatum*, pangenomic study, virulence factors, CRISPR types, secondary metabolite biosynthetic gene clusters

## Abstract

Fusobacterium nucleatum is a prevalent periodontal pathogen and is associated with many systemic diseases. Our knowledge of the genomic characteristics and pathogenic effectors of different F. nucleatum strains is limited. In this study, we completed the whole genome assembly of the 4 F. nucleatum strains and carried out a comprehensive pangenomic study of 30 strains with their complete genome sequences. Phylogenetic analysis revealed that the F. nucleatum strains are mainly divided into 4 subspecies, while 1 of the sequenced strains was classified into a new subspecies. Gene composition analysis revealed that a total of 517 “core/soft-core genes” with housekeeping functions widely distributed in almost all the strains. Each subspecies had a unique gene cluster shared by strains within the subspecies. Analysis of the virulence factors revealed that many virulence factors were widely distributed across all the strains, with some present in multiple copies. Some virulence genes showed no consistent occurrence rule at the subspecies level and were specifically distributed in certain strains. The genomic islands mainly revealed strain-specific characteristics instead of subspecies level consistency, while CRISPR types and secondary metabolite biosynthetic gene clusters were identically distributed in F. nucleatum strains from the same subspecies. The variation in amino acid sites in the adhesion protein FadA did not affect the monomer and dimer 3D structures, but it may affect the binding surface and the stability of binding to host receptors. This study provides a basis for the pathogenic study of F. nucleatum at the subspecies and strain levels.

**IMPORTANCE** We used F. nucleatum as an example to analyze the genomic characteristics of oral pathogens at the species, subspecies, and strain levels and elucidate the similarities and differences in functional genes and virulence factors among different subspecies/strains of the same oral pathogen. We believe that the unique biological characteristics of each subspecies/strain can be attributed to the differences in functional gene clusters or the presence/absence of certain virulence genes. This study showed that F. nucleatum strains from the same subspecies had similar functional gene compositions, CRISPR types, and secondary metabolite biosynthetic gene clusters, while pathogenic genes, such as virulence genes, antibiotic resistance genes, and GIs, had more strain level specificity. The findings of this study suggest that, for microbial pathogenicity studies, we should carefully consider the subspecies/strains being used, as different strains may vary greatly.

## INTRODUCTION

Fusobacterium nucleatum is a Gram-negative, spindle, non-spore-forming anaerobic bacterium belonging to the phylum *Fusobacterium* and family *Bacteroidaceae* (1). As a prevalent oral pathogenic bacterium, F. nucleatum can promote the occurrence and development of periodontitis (2, 3). Recent studies have shown that F. nucleatum can translocate to and colonize multiple human tissues and organs through a variety of strategies (4, 5) and contribute to the development of many systemic diseases, such as atherosclerosis, diabetes mellitus, pregnancy complications, premature delivery, respiratory infectious diseases, and Alzheimer's disease (6–10). Moreover, F. nucleatum plays an important role in the development and metastasis of many cancer types, including head and neck squamous cell carcinoma (11, 12), colorectal cancer (13, 14), breast cancer, and esophageal cancer (15), etc. Adhesion is one of the important strategies by which F. nucleatum exerts its pathogenicity toward the host, and among adhesion-related proteins, FadA (16, 17), Fap2 (18, 19), FomA (20), and RadD (21, 22) have been well-studied, but our knowledge of their association with pathogenicity-related genes and their pathogenesis strategies is still far from sufficient.

Evolutionary and phylogenetic analysis have shown that F. nucleatum strains can be mainly grouped into 4 subspecies, namely, *nucleatum*, *polymorphum*, *vincentii* (*fusiforme*), and *animalis* (23–28). Each subspecies prefers to inhabit certain body sites and induce inflammation-related diseases in different ways. Specifically, subsp. *polymorphum* and *vincentii* are frequently isolated from the mouths of healthy individuals (26, 29), while subsp. *nucleatum* is enriched in the focal sites of periodontal diseases (26, 29) and is associated with a variety of oral and nonoral infections (30). Subsp. *animalis* is frequently found in the colon (26) and can induce inflammation and monocyte activation in the human intestinal mucosa and promote the development of colorectal cancer (31, 32). In addition, a study revealed that subsp. *vincentii* promotes chronic prostate inflammation and associated with benign prostatic hyperplasia and prostatic cancer (33). This suggests that different subspecies may have significantly different genetic characteristics.

The first genomic study of F. nucleatum was reported in 2002 and revealed the metabolic characteristics of organic acid, amino acid, carbohydrate, and lipid in strain ATCC 25586 (34). A comparative genomic study on F. nucleatum strains showed that the antibiotic and other general drug resistance varied greatly among strains (35), subsp. *polymorphum* is more adept at horizontal gene transfer (HGT) than subsp. *nucleatum* and *vincentii* (36). Some newly discovered strains, such as W1481, do not belong to any of the 4 classic subspecies (37, 38). However, pangenomic studies of the functional characteristics and differences among F. nucleatum strains are still rarely reported.

In this study, we completed the genome maps of four different F. nucleatum strains isolated from our oral microbial library, followed by a comparative analysis of 30 F. nucleatum strains with complete genomic data. We aimed to reveal the distribution of functional genes, antibiotic resistance genes, and virulence genes at the subspecies/strains level to better understand the unique biological characteristics of F. nucleatum.

## RESULTS

### The phylogenetic analysis of F. nucleatum reveals a new subspecies strain, FNU.

**FIG 1:**
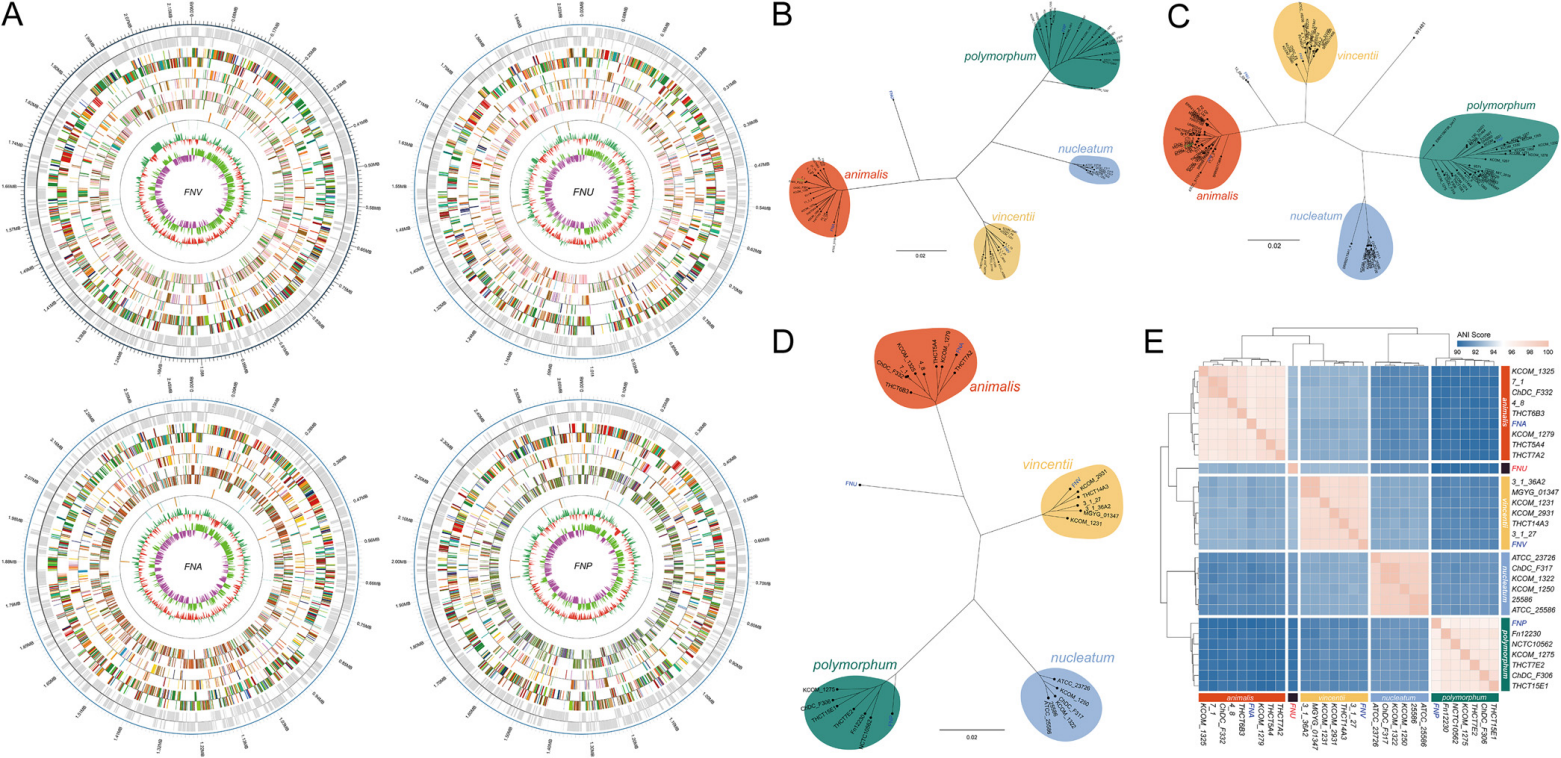
The complete genome map and phylogenetic tree of F. nucleatum strains. (A) The complete genome map of strains *FNA*, *FNV*, *FNP*, and *FNU*. The outermost circle is the position coordinates of genome sequence. From outside to inside, there are coding genes, gene function, ncRNA and GC content. (B) The phylogenetic tree of 59 F. nucleatum strains (4 sequenced strains and 55 NCBI download strains). (C) The phylogenetic tree of 4 sequenced strains and all the NCBI available F. nucleatum strains. (D) The phylogenetic tree of 30 strains (26 strains NCBI downloaded and 4 sequenced strains). (E) ANI analysis of 30 F. nucleatum strains.

We obtained 4 strains of F. nucleatum and carried out high-throughput sequencing, assembled the DNA sequences, and obtained the complete genome map of each strain (Materials and Methods) (Fig. 1A and Table 1). We constructed a phylogenetic tree with 858 single-copy orthologous proteins obtained from 4 sequenced strains and 55 strains of F. nucleatum in the NCBI database (Fig. 1B, and Table S1) and showed that most of the strains belonged to 1 of 4 subspecies: *nucleatum*, *polymorphum*, *vincentii* (*fusiforme*), and *animalis*. Three of the sequenced strains, named FNV, FNA, and FNP, were classified into subsp. *vincentii*, *animalis*, and *polymorphum*, respectively. However, 1 new strain (FNU) could not be classified into any of these subspecies. Furthermore, we selected *Fusobacterium periodonticum* (*F. periodonticum*) and *Fusobacterium hwasookii* (*F. hwasookii*) as outgroups to construct a phylogenetic tree with F. nucleatum (Fig. S1). The results showed that sequence similarity between *F. hwasookii* strains and F. nucleatum subsp. *polymorphum* was much higher than those between different F. nucleatum subspecies.

**TABLE 1:** The genomic information of strain FNV, FNU, FNA and FNP

Strain	FNV	FNU	FNA	FNP
Subspecies	*vincentii*		*animalis*	*polymorphum*
Size (bp)	2,194,806	2,057,857	2,492,833	2,651,846
GC (%)	27.41	27.13	27.08	26.98
tRNA	47	47	46	47
rRNA	15	15	15	15
CDs	2,003	1,934	2,346	2,566
No. of Reads	70,474	29,759	54,580	69,914
Mean Read Length (bp)	9,180	6,593	8,095	8,166
N50 Read Length (bp)	11,166	7,531	10,407	9,440
Polished Contigs	1	1	1	1
Max Contig Length (bp)	2,218,024	1,706,544	2,503,781	2,663,886
N50 Contig Length (bp)	2,218,024	1,706,544	2,503,781	2,663,886
Sum of Contig Lengths (bp)	2,218,024	2,519,044	3,954,673	5,306,697

To determine whether FNU could be classified as a new subspecies, a phylogenetic tree was constructed using 39 single-copy orthologous proteins obtained from four sequenced strains and all F. nucleatum strains in the NCBI database (table S1). The phylogenetic tree showed the evolutionary interrelations and classification of the four strains, and FNU formed an independent evolutionary branch with the strain 13_08_02 (Fig. 1C). Forty-two F. nucleatum strains without previous subspecies annotation were also annotated to their corresponding subspecies (Table S1). Next, 30 F. nucleatum strains with complete genome information (26 strains from the NCBI database and the 4 strains that we sequenced) were used to carry out phylogenetic analysis and an average nucleotide identity (ANI) analysis. The results showed that FNU was in a new branch (Fig. 1D and E, and Table S1 and 2), which located between subsp. *animalis* and subsp. *vincentii*. All these results suggest that FNU is a representative strain of a new F. nucleatum subspecies, and the genomic characteristics of strain FNU are shown in Table 1.

### The distribution of core/soft-core and subspecies-unique genes.

**FIG 2:**
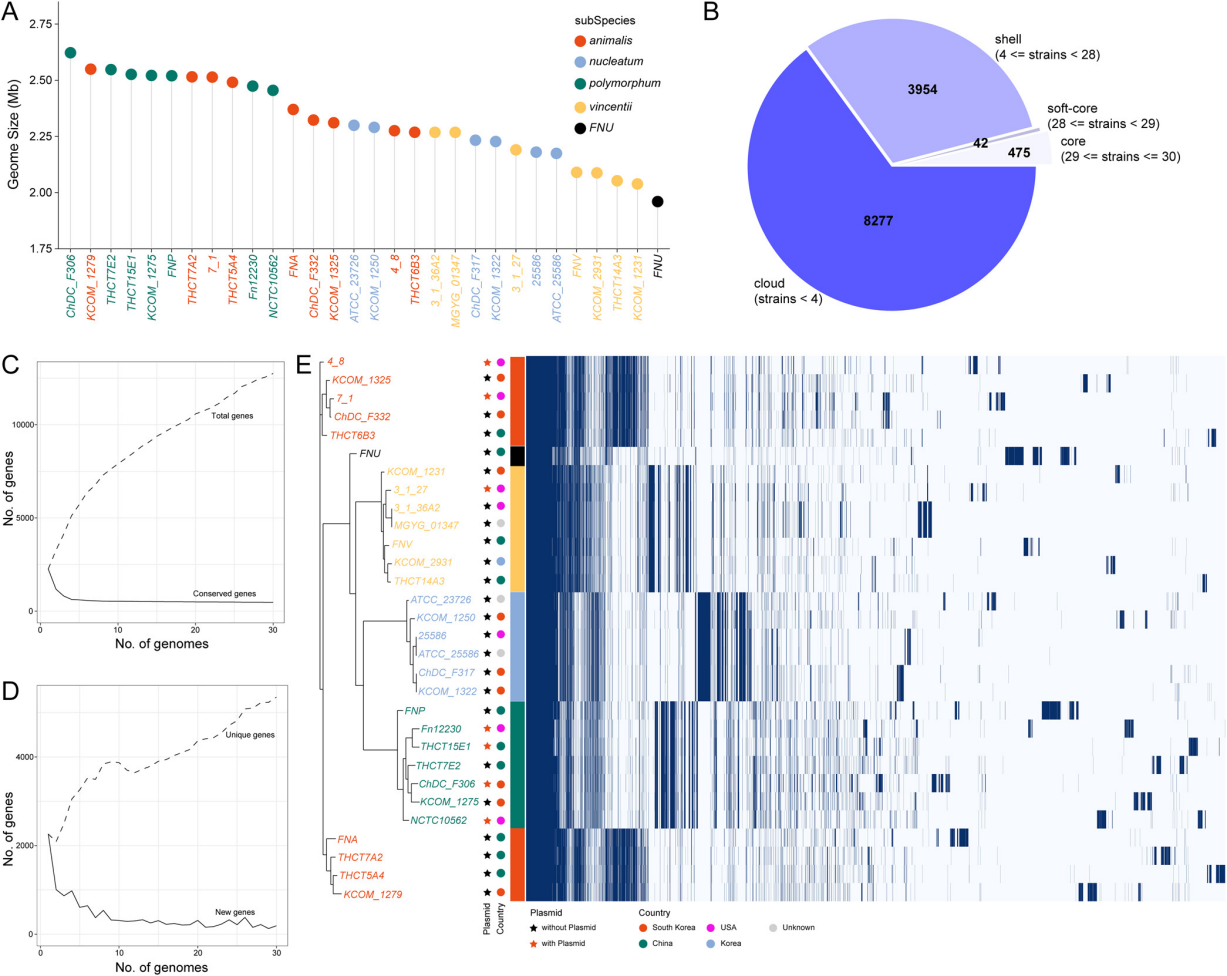
The pan-genomic information of 30 F. nucleatum strains. (A) Distribution of genome size among 30 strains. (B) Number of core genes, softcore genes, shell genes and cloud genes. (C) Curves of conserved and total genes. (D) Curves of new and unique genes. (E) The distribution of genes among 30 strains. Gene matrix shows the presence in blue and absence in white. The dendrogram is the phylogenetic relationship in 30 F. nucleatum strains. Plasmid presence, number, and isolation location of 30 F. nucleatum strains.

We carried out pangenomic analysis of 30 F. nucleatum strains with a complete genome map. Fig. 2A showed that subsp. *polymorphum* and *animalis* had large genome sizes among the 30 strains, while the genome of subsp. *vincentii* was relatively small and that of FNU was the smallest. Regarding gene frequency, 475 core genes (29 <= strains < 30), 42 soft-core genes (28 <= strains < 29), 3,954 shell genes (4 <= strains < 28) and 8,277 cloud genes (strains < 4) were identified across the 30 strains (Fig. 2B). Pangenomic analysis showed that the number of conserved genes remained stable as the addition of total gene number increased (Fig. 2C), and the number of new genes also remained relatively stable as the number of unique genes increased (Fig. 2D), suggesting that the pangenome of F. nucleatum exhibited a closed pattern. We assessed the gene distribution characteristics among all the strains and reclustered them according to the pangenomic analysis results. The results revealed that the core and soft-core genes were shared by almost all the strains (Fig. 2E). A group of genes was uniquely shared among strains from the same subspecies (Table S3, and Fig. 2E). In addition, a large number of genes were specifically distributed in certain specific strains (Fig. 2E). These results suggest that the functional characteristics of F. nucleatum are conserved at the subspecies level and are specific to each strain.

Among the plasmids, there was no subspecies level specificity for the presence and number of plasmids and the separation locations among the 30 strains. For instance, 7 F. nucleatum strains, namely, subsp. animalis 4_8, *animalis* 7_1, *vincentii* 3_1_27, *polymorphum* Fn12230, *polymorphum* THCT15E1, *polymorphum* ChDC F306, and *polymorphum* NCTC10562 comprised of different number of plasmids (Fig. 2E). We constructed a phylogenetic tree of 7 F. nucleatum strains containing plasmids (Fig. S2). The results showed that the relationship between subsp. *animalis* 4_8 and subsp. *polymorphum* ChDC_F306 might be closer. There are shared plasmid elements. We carried out gene annotation and gene similarity analysis on all plasmids using the OrthoFinder (version 2.2.6) software and found that multiple genes were shared in the plasmids of different strains (Table S8 plasmid). We found that there were no virulence genes and AMR-associated genes contained in any of these plasmids.

### Functional annotation of core/soft-core and subspecies-unique genes.

**FIG 3:**
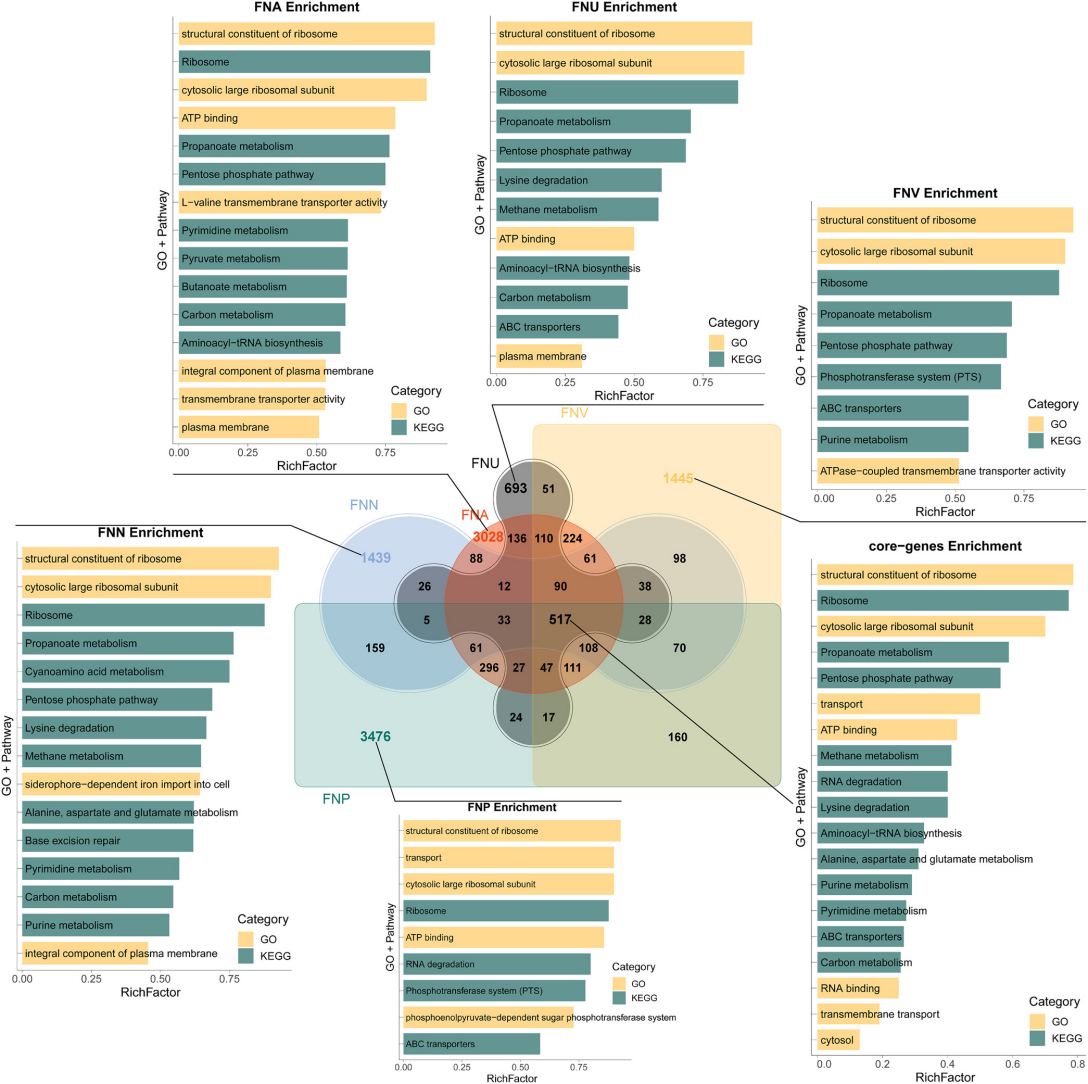
Functional analysis of core genes and subspecies-unique genes.

The functions of core/soft-core and subspecies-unique genes were annotated with the GO biological progress and KEGG pathway database, and the results showed that most of the 517 core/soft-core genes were housekeeping genes, that were annotated to structural constituents of ribosomes, cytosolic large ribosomal subunits, ATP binding, propanoate metabolism, and the pentose phosphate pathways. The subspecies-unique genes were more closely related to corresponding unique biological behavior and characteristics. Specifically, subsp. *animalis*-unique genes were associated with membrane composition and transmembrane transporter-related terms, such as integral component of the plasma membrane and transmembrane transporter activity (Fig. 3). Subsp. *nucleatum*-unique genes were related to the siderophore-dependent iron import into the cell, integral components of the plasma membrane and structural constituents of ribosomes (Fig. 3). Subsp. *polymorphum*-unique genes were annotated to the phosphotransferase system (PTS) and phosphoenolpyruvate-dependent sugar phosphotransferase system (Fig. 3). The PTS can regulate biofilm formation and colonization by pathogenic bacteria and may play an important role in the pathogenicity of F. nucleatum. Subsp. *vincentii*-unique genes were associated with ATPase-coupled transmembrane transporter activity, ABC transporters, and the PTS, etc. FNU-unique genes were annotated to structural constituents of ribosomes, propanoate metabolism, the pentose phosphate pathway, and ABC transporters, etc. (Fig. 3). Since FNU was the only strain with a complete genome in this new subspecies, the function analysis of this subspecies was inadequate.

### The distribution of virulence factors, antibiotic resistance genes, and secretion systems.

**FIG 4:**
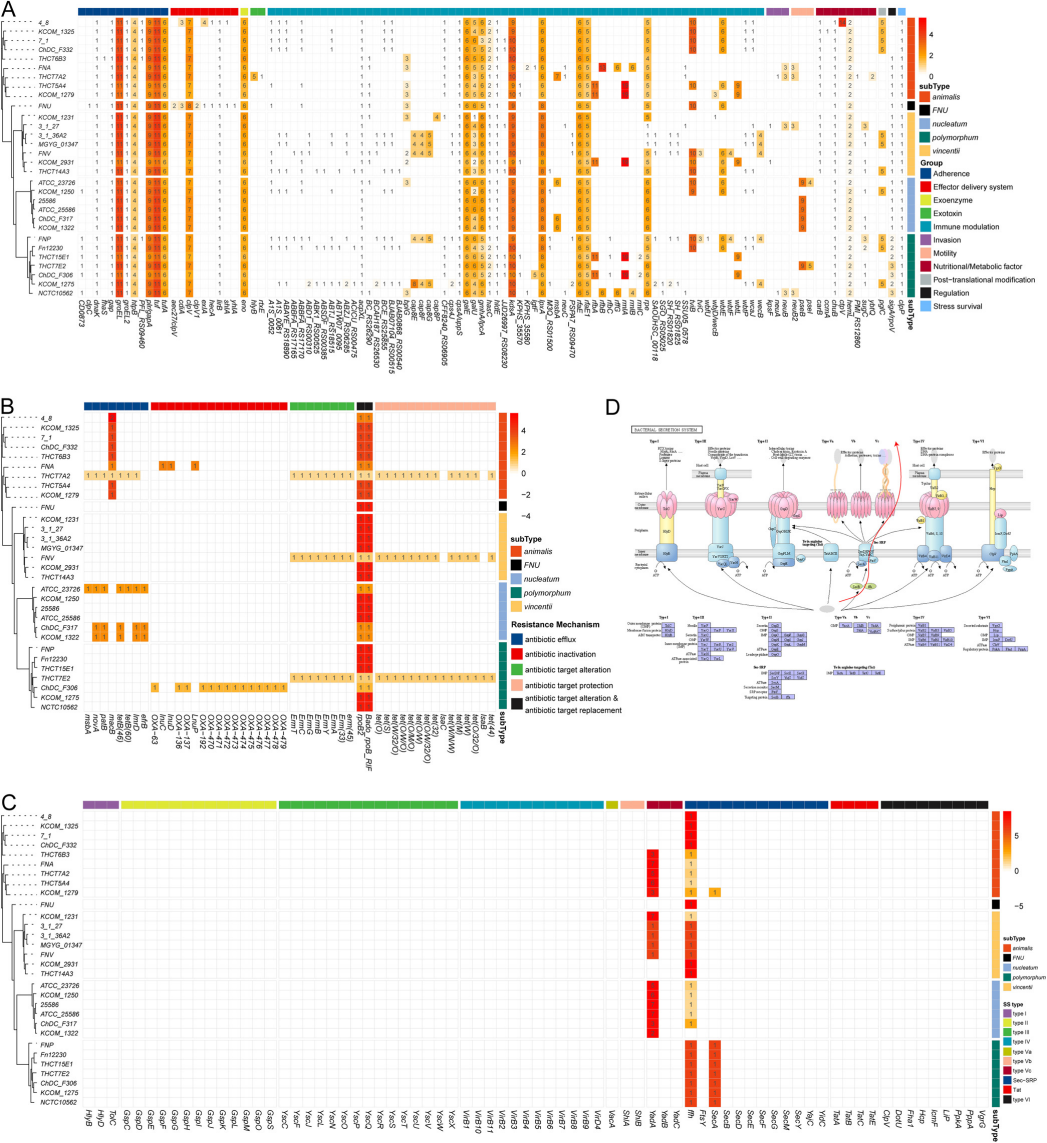
Distribution of virulence genes, antibiotic resistance genes, and protein secretion system. (A to C) Distribution of virulence gene, antibiotic resistance genes, and protein secretion system. Number represents the copy of corresponding genes. (D) Diagram of bacterial secretion system. Red arrow represents the secretory system of F. nucleatum.

To explore the potential pathogenicity of the F. nucleatum, the Virulence Factor Database (VFDB) was used to define virulence factors (VFs) in 30 strains. Globally, 107 VFs from 11 virulence categories were detected in the genome of F. nucleatum (Fig. 4A, Table S4). In particular, a variety of adhesion-related VFs, such as *groEL*, *plr/gapA*, *tuf*, *tufa*, and *htpB*, were detected in all strains with multiple copies, which suggests that adhesion is an important factor in the pathogenicity of F. nucleatum. Of the VFs related to the effector delivery system, *clpV* was present in all strains and maintained a high copy number (7 copies) (Fig. 4A). VFs associated with immune modulation, *galU*, *galE*, *gmhA*/lpcA, *kdsA*, *lpxA*, *rfaE*, *rfaE1*, and *rpe*, were also present in all the strains with multiple copies (Fig. 4A). In addition, there were several VFs with low copy numbers (1 or 2) that were widely distributed among all the strains.

Notably, many VFs showed no consistent occurrence rule at the subspecies level. For example, *cdiA* and *exlA* were present in subsp. *animalis* 4_8 and FNU; *cap8P* only resided in subsp. *vincentii* KCOM 1231; *wbfU* could be detected in only subsp. *polymorphum* FNP; and *ctpV*, which is associated with nutritional/metabolic factors, was present in only subsp. *animalis* 4_8 (Fig. 4A). These results indicate that the pathogenesis of F. nucleatum strains may be strain specific.

The Comprehensive Antibiotic Resistance Database (CARD) was used to identify antibiotic resistance genes (ARGs) among F. nucleatum strains, and the ARG distribution also showed similar strain-specific characteristics (Fig. 4B, Table S5). The genes *rpoB2* and *Bado_rpoB_RIF*, which confer resistance to rifamycin, were detected in all the strains (Fig. 4B). The *OXA* family genes, which confer resistance to carbapenem, cephalosporin, and penam, were enriched in subsp. *polymorphum* ChDC F306. The *Eme* and *tet* family genes were mainly enriched in strain of subsp. *animalis* THCT7A2, *vincentii* FNV, and *polymorphum* THCT7E2. FNU carried the ARGs common to all the strains. These results suggest that strain-specific information on ARGs should be carefully considered in the treatment of F. nucleatum-induced diseases.

In contrast to VFs and ARGs, the secretion system-related genes showed no specificity at the subspecies or strain level (Fig. 4C and D). The genes *ffh* and *YadA* had identical distribution patterns, while *SecA* was mainly detected in subsp. *polymorphum*.

### The distribution of genomic islands and CRISPR types.

**FIG 5:**
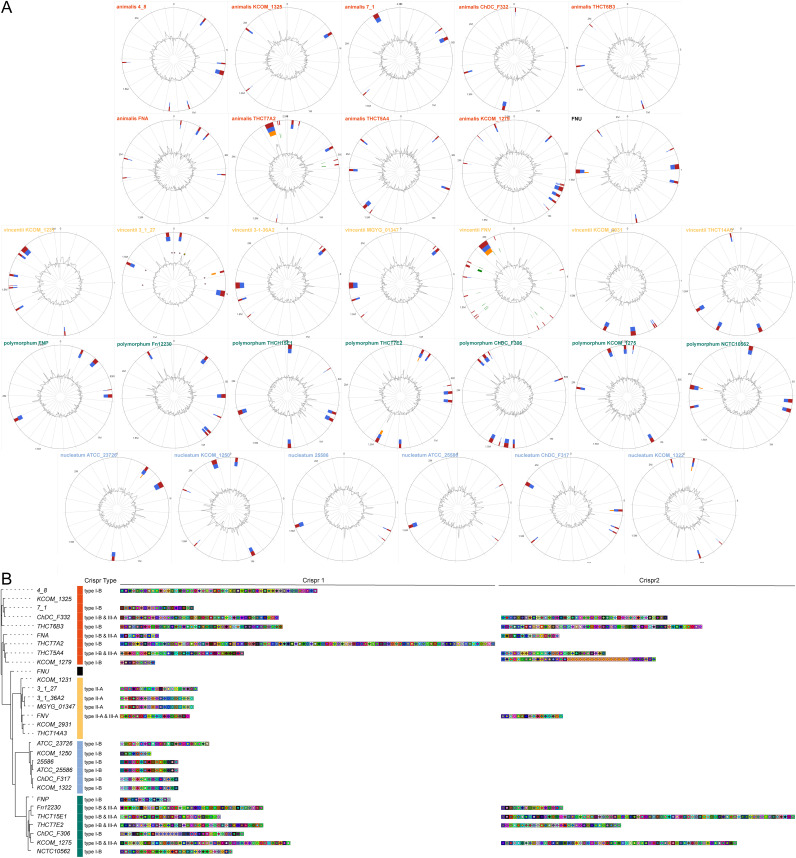
GIs and CRISPR types. (A) GIs of 30 F. nucleatum strains. The blocks on the inner ring are colored according to the tool used for prediction, IslandPick (green), IslandPath-DIMOB (blue), SIGI-HMM (orange), and the integrated results (dark red). The light pink circle represents an antibiotic resistance gene from the genome island and the yellow circle represents a pathogen-related gene (yellow circle). (B) The CRISPR types of F. nucleatum strains.

We used IslandViewer based on the IslandPick, IslandPath-DIMOB, SIGI-HMM and Islander databases to reveal the characteristics of genomic islands (GIs) among F. nucleatum strains. Fig. 5A showed that GIs tended to distribute strain-specifically, and the location of GIs was also strain specific (Table S6). Each strain contained an average of 6 GIs, while strain FNV had the highest number of GIs at 15, subsp. *nucleatum* 25586 and *animalis* KCOM 1325 had only 3.

Functional annotation showed that GIs from different subspecies/strains carried genes with different functions. Specifically, GIs in subsp. *animalis* and *polymorphum* contained genes encoding a recombinant protein, the murein hydrolase activator *EnvC*, a NAD kinase, the DNA repair protein *RecN* and the tyrosine recombinase *XerD*. CRISPR-associated endoribonuclease/proteins were identified in *vincentii* FNV, *animalis* KCOM 1279, and *animalis* THCT7A2. Type II secretion system proteins were present in *vincentii* 3_1_27, and *vincentii* KCOM_1231, etc. Toxin zeta was found in *vincentii* 3_1_27, *animalis* 4_8, *animalis* 7_1, *animalis* KCOM_1325, etc. Antitoxin HigA/YwqK was found in *vincentii* KCOM 2931, *vincentii* KCOM_1231, etc. PTS system component was identified in *vincentii* FNV, *vincentii* KCOM 2931, and *vincentii* THCT14A3, etc.

Clustered regularly interspaced short palindromic repeat (CRISPR) is an evolving and adaptive immune defense element that widely distributes in bacteria and archaea. We used CRISPRCasFinder and CRISPRDetect to classify CRISPR sequences among the 30 F. nucleatum strains (Table S7). The results showed that strains from the same subspecies had identical CRISPR types (Fig. 5B). For example, strains from subsp. *animalis*, *nucleatum* and *polymorphum* mainly had CRISPR types I-B and III-A, while strains from subsp. *vincentii* mainly carried CRISPR type II-A. There was a certain consistency in spacer types within subspecies, while the quantity was strain-specific (Fig. 5B).

### The distribution of secondary metabolite biosynthetic gene clusters and prophages.

**FIG 6:**
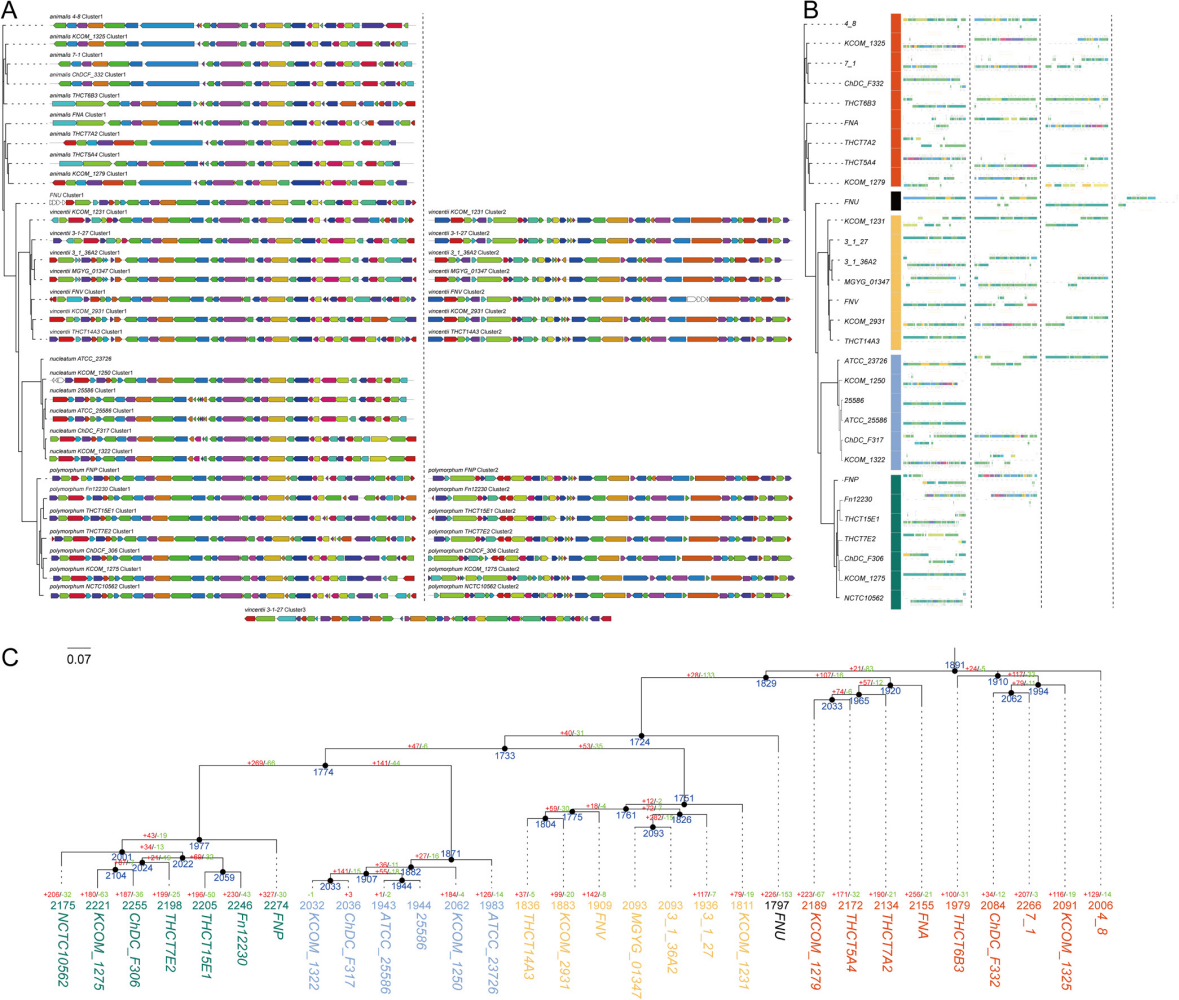
Secondary Metabolite Biosynthetic Gene Clusters and prophages. (A) Distribution of Secondary Metabolite Biosynthetic Gene Clusters. (B) The type and number of prophage sequences. (C) Distribution of gene families in F. nucleatum strains.

The secondary metabolite (SM) biosynthetic gene clusters in F. nucleatum strains were annotated using the antiSMASH database. As shown in Fig. 6A, the main secondary metabolite (SM) in F. nucleatum was synthesized by nonribosomal peptide synthetase (NRPS), which is a biosynthetic enzyme that produces various nonribosomal peptides and can be widely used in clinical applications, for example, as antibiotics, antitumor inhibitors, and immune-suppressants (39, 40). The NRPS was subspecies-specific in terms of gene composition, gene number, gene arrangement, and transcriptional direction. As shown in the evolutionary tree, subsp. *animalis* appeared first, followed by subsp. *vincentii* and finally *nucleatum* and *polymorphum* (Fig. 6A). From subsp. *animalis* to *vincentii*, F. nucleatum experienced the expansion of gene families, while from subsp. *vincentii* to *nucleatum* and *polymorphum* experienced the retention and loss of gene families (Fig. 6C).

A prophage is the nucleic acid sequence of a mild bacteriophage integrated into the host genome. This sequence contains certain functional genes and can be transferred between microorganisms. We annotated the prophages with PHASTER and showed that prophage sequences were prevalent in all F. nucleatum strains (Fig. 6B), indicating that all these 30 bacterial strains may be lysogenic bacteria. However, the type and number of prophage sequences and their location and orientation on the genome, were strain specific.

### Variations in FadA may impact its combination mode with the host receptor.

**FIG 7:**
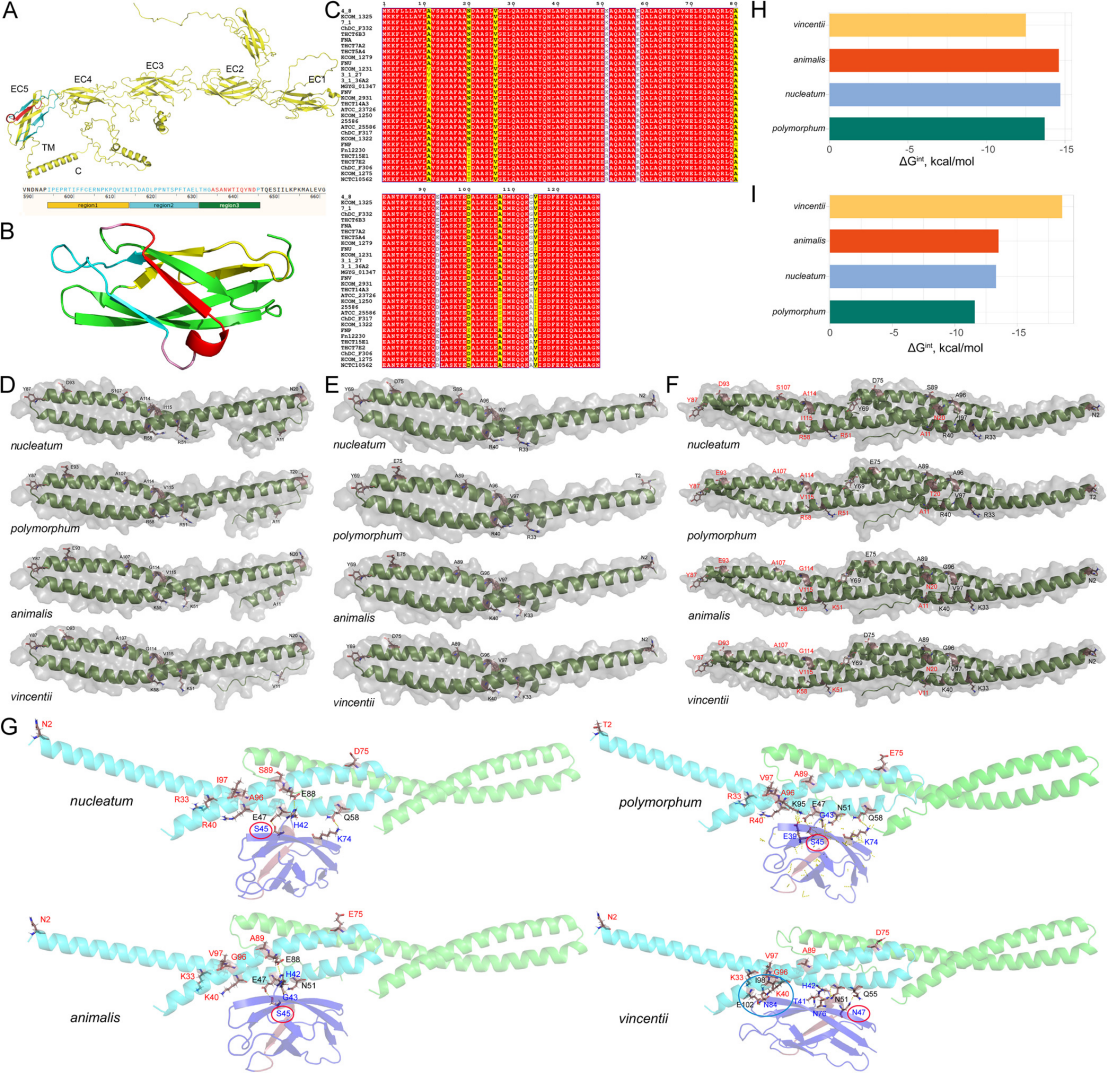
Structure diagrams of FadA and combining mode of FadAc and E-cadherin in different subspecies. (A) 3D protein structure diagram of E-cadherin. EC5 is the region(red) of E-cadherin binding to FadA. (B) The expanded diagram of EC5 domain. Green is region 1, blue is region 2, and red is region 3 (FadA binding location). (C) The amino acid sequence of FadA in30 strains. Yellow and white indicate the mutation sites. (D to F) Structure of pre-FadA, mFadA and FadAc. (G) The 3D protein structure diagram of FadAc and E-cadherin. (H) and (I) The binding energy of pre-FadA to mature FadA, and FadAc to E-cadherin.

Adhering to and invading host cells are important ways by which pathogenic bacteria to exert pathogenicity and escape immune surveillance (41–43). Studies have shown that F. nucleatum can exert its pathogenicity through the adhesion protein FadA (5, 44, 45). FadA can adhere to the EC5 domain of the surface receptor E-cadherin on colon cancer cells (16). Using AlphaFold2, we predicted the detailed structure of E-cadherin and EC5 (Fig. 7A and B). With the application of mafft, we found 11 amino acid sites in FadA mutated with subspecies specificity (Fig. 7C). Therefore, FadA protein sequences of 4 main subspecies were selected as representatives for further analysis. The main structure of precursor FadA (pre-FadA) was consistent across all subspecies, even though the amino acids at many sites varied (Fig. 7D). FadA works through the combination of pre-FadA and mature FadA (mFadA) to form a pre-FadA-mFadA complex (FadAc), which can combine with the host receptor protein E-cadherin (16, 45, 46). We predicted the structure of mFadA by AlphaFold2, the predicted structure was consistent with the reported crystal structure (47), and Y69 was the turning point in the hairpin loop (Fig. 7E). Although FadA had multiple mutation sites in different subspecies, the binding mode of pre-FadA and mFadA remained the same among the 4 subspecies, independent of mutation sites (Fig. 7F).

Subsequently, the index of the protein-protein interaction (PPI) surface was analyzed using PDBePISA. Fig. 7H showed that subsp. *animalis* and *nucleatum* released more energy to form FadAc than the other 2 subspecies. Next, the FadAc and EC5 complex structures were determined by AlphaFold2 and the binding region of EC5 with FadAc was labeled in red. As shown in Fig. 7A and G, the identified binding region of EC5 with FadAc was consistent with the experimental study (16). The binding mode of subsp. *vincentii* was different from other subspecies, with the largest interface area and lowest binding free energy (Fig. 7G and I). Then, we applied the steered molecular dynamics (SMD) to calculate the free energy information of the interaction between E-cadherin and FadA. The result obtained from the analysis with SMD is consistent with the study with PDBePISA (Fig. S3).

## DISCUSSION

F. nucleatum is a well-known pathogen associated with periodontitis and multiple systemic diseases, but knowledge of the genomic characteristics of different F. nucleatum strains is limited. In this study, we performed a pangenomic analysis of 30 F. nucleatum strains, providing a fundamental basis for the pathogenesis research and clinical treatment. The phylogenetic tree obtained for multiple protein units in this study was consistent with the results of previous studies. Similar to W1481 (38), the newly sequenced strain FNU was proven to be a new subspecies by multiple phylogenetic tree analysis and ANI analysis, which was located between subsp. *animalis* and *vincentii.* In contrast, our study categorized strain F0401 as subsp. *animalis* by multiple phylogenetic tree analysis, while a previous study reported that F0401 may belong to subsp. *polymorphum* based on 1 phylogenetic tree (48).

Our study showed that each subspecies had unique gene clusters that were closely related to the biological characteristics of these subspecies. Therefore, we believe that this is the genetic basis for the strains of the same subspecies having more similar biological characteristics than strains from different subspecies. This study may provide new insights for identifying similar biological characteristics among F. nucleatum strains from the same subspecies.

The unique gene-signatures were also observed at the strain level. For instance, some VFs were strain specific, such as ARGs, the location and function of GIs, and the number of prophages. As some of the pathogenic genes varied among F. nucleatum strains, it is necessary to identify the unique virulence genes from corresponding strains specifically responsible for certain diseases. For treatment of diseases induced by F. nucleatum, the strain-specific ARGs should be considered.

CRISPR provides acquired immunity against foreign genetic elements for bacteria. In this study, we systematically analyzed the distribution of CRISPR types among 30 F. nucleatum strains and found that strains from the same subspecies had identical CRISPR types. Subsp. *animalis*, *nucleatum*, and *polymorphum* contain CRISPR type I-B, which is widely used for efficient genome editing in *Clostridium* and the Cas protein can use certain crRNAs to mediate immunity during recurring viral infection (49, 50).

In this study, we proved that FNU belongs to a new subspecies. FNU maintained the smallest genomic size among 30 F. nucleatum strains, and the genes were mainly annotated as housekeeping genes, such as genes related to ribosomes, cytosolic large ribosomal subunits, ATP binding, carbon metabolism, and ABC transporters. While virulence factors, such as *clpc*, *cdiA*, *aec27/clpV*, *clpV1*, and *yhvA*, existed only in FNU, which suggests that FNU may contain some unique pathogenic characteristics. FNU only contained the common ARGs *rpoB2* and *Bado_rpoB_RIF*. The distribution preference of this strain in the human body and the pathogenic characteristics of the strain needs further study.

The phylogenetic analysis in Fig. 6C shows that subsp. *animalis* evolved first, followed by subsp. *vincentii*, and finally *nucleatum* and *polymorphum*. We have also analyzed the gain and loss of gene families in the 30 F. nucleatum strains (Fig. 6C). However, since there is no fossil node between F. nucleatum and its near-source species, reliable evolutionary time analysis could not be performed.

In summary, this study showed the differences in the genomic characteristics of F. nucleatum subspecies among 30 strains, indicating that classifying bacterial strains from the same species into certain subspecies is of great significance for obtaining an in-depth understanding of their functional characteristics. Studies on the pathogenesis, antibiotic resistance, and clinical treatment of F. nucleatum should carefully consider the genomic characteristics specifically at the subspecies and strain levels.

## MATERIALS AND METHODS

### F. nucleatum isolation and culture.

We obtained 4 strains of F. nucleatum from the oral microbial resource library of our laboratory based on PCR amplification and Sanger sequencing of 16S rRNA sequences. F. nucleatum was inoculated on brain-cardio-perfusion (BHI) solid medium (BHI liquid medium, agar powder, 5% sterile defibrin sheep blood, 0.1% heme chloride, and vitamin K) and cultured in anaerobic incubators (80% N_2_, 10% H_2_, and 10% CO_2_) at 37 degrees C (37°C anaerobic incubators) for 72 h. Then, single colonies were selected and placed in BHI liquid medium for proliferation to the logarithmic growth stage. The cultures were then centrifuged at 4500 rpm for 5 min to collect the bacterial cell, which were re-suspended in BHI liquid medium for subculture. The purity of the bacteria was determined by PCR and 16S rRNA sequencing. The bacterial cell culture methods were performed as described previously.

### Genome sequencing and assembly.

Genomic DNA was extracted by the SDS method (51). The detection and quality of the isolated DNA samples were evaluated using agarose gel electrophoresis and a Qubit 2.0 Fluorometer (Thermo Scientific). The PacBio Sequel and Illumina NovaSeq platforms were used to construct the DNA library. The whole genomes of these 4 strains (subsp. *vincentii* FNV, FNU, subsp. *animalis* FNA, and subsp. *polymorphum* FNP) were sequenced using the PacBio Sequel platform and Illumina NovaSeq PE150 at the Beijing Novogene Bioinformatics Technology Co., Ltd. The whole genome sequences of the four strains were deposited as a BioProject (PRJNA932162) in GenBank under the accession numbers CP117525 (FNV), CP117526 (FNU), CP117797 (FNA), and CP117825 (FNP).

### Data acquisition and gene annotation.

In addition to the 4 newly sequenced strains, the genomes of all the F. nucleatum strains available in the NCBI database were downloaded. The genomic information of F. nucleatum is summarized in Table S1. We downloaded the genome sequences of F. periodonticum and *F. hwasookii* from the NCBI, whose detailed information was summarized in Table S1. Then, all the downloaded genomic nucleic acid sequences were annotated by Prokka software (version 1.12) (52) with default parameters. Of these strains, 55 strains had clear subspecies information and were used for subspecies annotation analysis of other strains, and 26 strains had complete genome maps and were selected for further comparative genomic analysis.

### Protein family analysis and phylogenetic analysis.

Based on the Prokka annotation results, we obtained the coding protein sequences of each F. nucleatum strain. Protein family analysis was performed by OrthoFinder software (version 2.2.6) (53) with default parameters. Then, we performed phylogenetic analysis to construct an evolutionary tree with the single-copy lineal homologous genes obtained by OrthoFinder. The single-copy lineal homologous genes of each strain were ordered and connected head to tail to form a long sequence.

The MAFFT software (version 7.407) (54) was used to perform the multisequence alignment with long single-copy lineal homologous sequences and GBlocks software (version 0.91b) (55) was used to extract the conserved sites from the results of the multisequence alignment with default parameters. Then, modeltest-ng software (56) was used to calculate the optimal amino acid replacement model of conserved sites and then RAxML-NG software (version 0.9.0) (57) was applied to construct the maximum likelihood (ML) phylogenetic tree using the optimal amino acid replacement model. FigTree software (version 1.4.4) (http://tree.bio.ed.ac.uk/software/figtree) was used to visualize the best ML tree result and analyze the phylogenetic relationships among different F. nucleatum strains and subspecies.

To further assess the boundaries between different subspecies of F. nucleatum, an ANI analysis was performed using the FastANI software (58) with default parameters, and the results were visualized as a heatmap.

### Pangenomic analysis.

Based on the gff files obtained from the Prokka annotation results, we used Roary software (59) to perform a pangenomic analysis of 30 F. nucleatum strains with complete genome maps. By Roary pangenome analysis, we obtained four different classes of genes: the 'core' (99% <= strains <= 100%), 'soft core' (95% <= strains < 99%), 'shell' (15% <= strains < 95%) and 'cloud' (0% <= strains < 15%) groups.

Next, we used the jveen tool (60) (http://jvenn.toulouse.inra.fr/app/example.html) to identify subspecies-specific gene families from the pangenomic analysis results. The representative sequence of each gene family was extracted by the Perl program and further annotated by KOBAS software (version 3.0.3) with the parameter -s: ko. Then, the R package clusterProfiler (version 4.4.4) was applied for GO and KEGG enrichment analysis based on the KOBAS annotation results of KOBAS. The statistically significant results (*P* < 0.05) of core gene families and subspecies-unique gene families were visualized in bar plots.

### Virulence genes and resistance genes annotation.

In this study, we extracted the virulence protein sequences from the VFDB to generate a blast reference database and downloaded the virulence protein sequences stored in VFDB as a reference and blasted them against the annotated protein sequences from the 30 F. nucleatum strains. To acquire optimal virulence gene analysis results, the blast e-value threshold was set to 1e-10, and the identity and query coverage were set to more than 50%. Following the above steps for virulence genes identification, CARD was used as a reference to annotate the optimal resistance genes in the 30 F. nucleatum strains.

### Identification of protein secretion systems.

We downloaded the available component protein sequences of all kinds of bacterial secretory systems from the KEGG database. The component protein sequences were used as references to perform the blast analysis. Using the methods, parameters and thresholds used in the virulence genes and resistance genes annotation process, we annotated the optimal secretory system component proteins in the 30 F. nucleatum strains by the BLASTP to identify the possible types of bacterial secretory systems.

### Genomic islands and CRISPR annotation.

IslandViewer is a commonly used genomic island prediction method that has 3 built-in precise prediction methods: Island Pick, Island Path-DiMob, and SIGI-HMM. IslandViewer predicts genomic islands by complementation of differences with built-in methods. In this analysis, genomic islands of F. nucleatum strains were predicted by IslandViewer (61). We uploaded the GBK-format files obtained by the Prokka software to the IslandViewer database and the consistent results predicted by multiple methods were selected as the optimal GIs in the corresponding genomes.

CRISPRFinder (62) and CRISPRDetect (63) with default parameters were used to identify CRISPR and detect the CRISPR types in F. nucleatum genomes. The relatively consistent results obtained by the 2 methods were regarded as reliable results and were visualized by CRISPRStudio (64).

### Secondary metabolite biosynthetic gene clusters and prophages.

The genome sequences of 30 F. nucleatum were uploaded to the antiSMASH (65) (version 6.11) database to predict the possible secondary metabolite biosynthesis gene clusters. Visualization of the results and comparative analysis of gene clusters were carried out by the multigeneBlast tool. Putative prophage annotation in each F. nucleatum strain was performed by the PHASTER (66) database.

### Gene gains and losses analysis.

Gene gains and losses have shaped the gene repertoire of species since the universal last common ancestor to species today. Genes in extant species were acquired at different historical time points via *de novo* creation of new genes, duplication of existing genes or transfer of genes from another species (HGT), and these genes are gradually lost (67). Based on the results of homologous gene analysis performed by OrthoFinder and phylogenetic analysis performed by Roary, the Count software (68) calculated the family history of F. nucleatum with Wagner parsimony.

### Comparative analysis of FadA.

From the Prokka annotation results, the amino acid sequences of FadA were extracted for the 30 F. nucleatum strains. MAFFT (version 7.407) was used to perform multiple sequence alignments of FadA sequences, and the results were visualized using ESPript 3 (69) (http://espript.ibcp.fr). Based on the multiple sequence alignment results, we selected a representative FadA sequence in each subspecies. AlphaFold2 (70) was used to predict the 3D coordinates of pre-FadA, mature FadA, E-cadherin, and FadAc.

PDBePISA is an interactive tool for the exploration of macromolecular interfaces (71, 72). Through the detailed calculation of the number and proportion of atoms on the interaction surface, the number and proportion of amino acids on the interaction surface, the interaction area and proportion, the solvation-free energy, the position and intensity of hydrogen bonds, disulfide bonds, salt bonds, and covalent bonds on the interaction surface, the evaluation indexes of the binding stability of the 2 proteins were finally obtained. With the computed results of PDBePISA (72), the stability of the complexes formed by pre-FadA, mature FadA, and E-cadherin was quantified.

To save running time, coarse-grained (CG) molecular dynamics simulation method was selected. It has been reported that CG molecular simulation can be used to accurately predict the strength of protein-protein interaction, while achieving a 500-times acceleration compared with all-atom molecular simulation (73). We used GROMACS for all MD simulations and MARTINI force field for CG simulations (74). The potential of mean force (PMF) was used to describe the strength of the interaction between 2 structures (75), and the calculation method of PMF was shown in reference (73). We chose the center of mass (COM) separation r as the reaction coordinate and repeated the equilibration procedure and the production runs at each distance, with 100 ns per production simulation run. In our MD simulation process, mature FadA and precursor FadA were treated as A protein, and E-cadherin was tested as B protein. Three MD runs were performed for each protein interaction. The following figure is the resulting PMF curve, where the dashed line represents the PMF curve for each independent MD and the solid line represents the mean PMF.

### Data availability.

The whole genome sequences of the four strains were deposited as a BioProject (PRJNA932162) in GenBank under the accession numbers CP117525 (FNV), CP117526 (FNU), CP117797 (FNA), and CP117825 (FNP).
