# Supplementary material for: Pangenomic Study of Fusobacterium nucleatum Reveals the Distribution of Pathogenic Genes and Functional Clusters at the Subspecies and Strain Levels
Source: Microbiol Spectr. 2023 Apr 12;11(3):e05184-22. doi: 10.1128/spectrum.05184-22 (PMC10269558; doi:10.1128/spectrum.05184-22)
Supplement: Supplemental file 9 — Fig. S1 to S3. Download spectrum.05184-22-s0008.pdf, PDF file, 0.3 MB [file spectrum.05184-22-s0008.pdf]

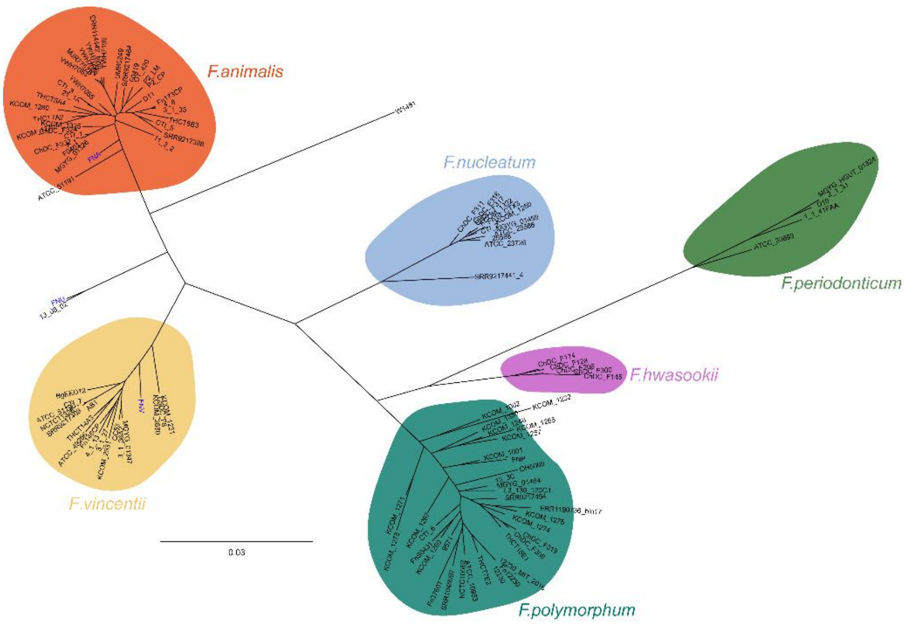

Supplementary figure 1 The phylogenetic analysis of *F. nucleatum*, *F. periodonticum* and *F. hwasookii*.

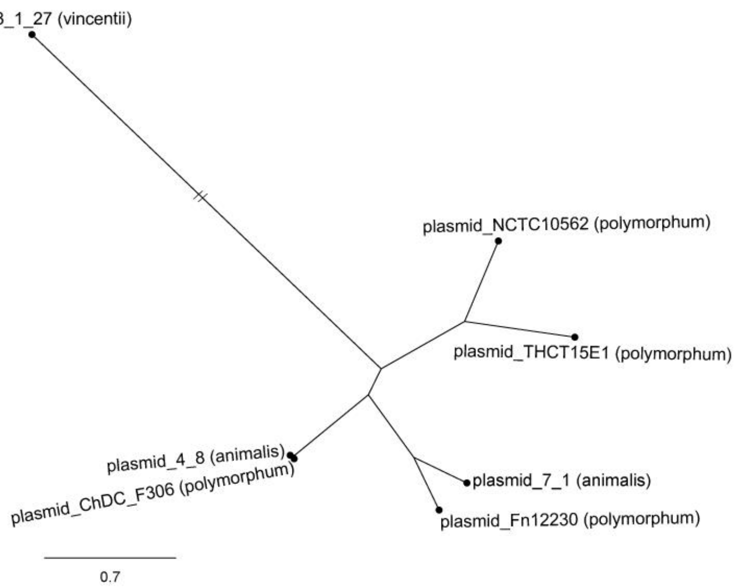

Supplementary figure 2 The phylogenetic tree of 7 *F. nucleatum* strains containing plasmids.

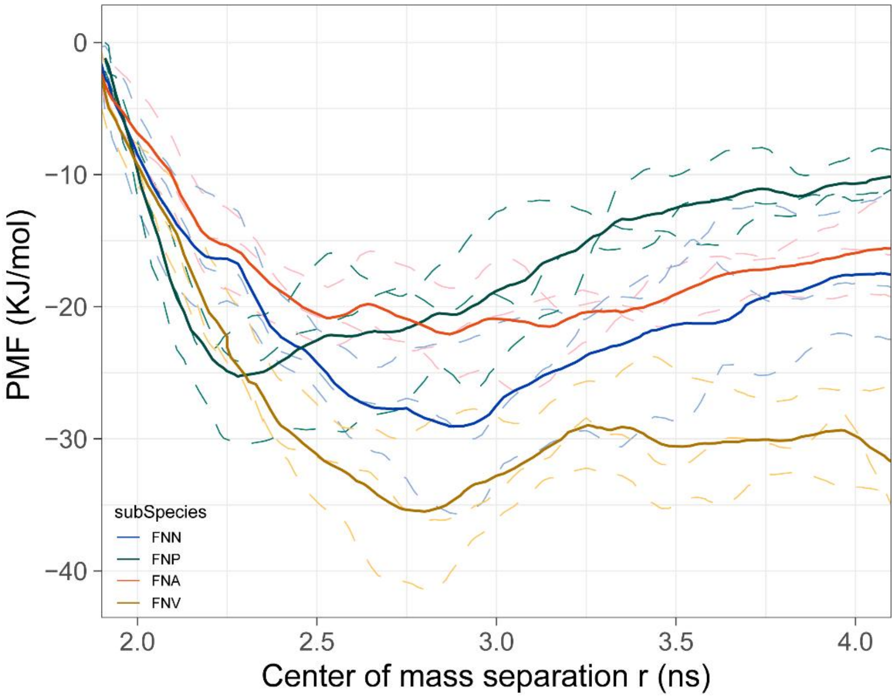

Supplementary figure 3 The steered molecular dynamics (SMD) calculate the free energy information of the interaction between E-cadherin and FadA.
